# *De novo* assembly and annotation of the Patagonian toothfish (*Dissostichus eleginoides*) genome

**DOI:** 10.1186/s12864-024-10141-4

**Published:** 2024-03-04

**Authors:** David Ryder, David Stone, Diana Minardi, Ainsley Riley, Justin Avant, Lisa Cross, Marta Soeffker, Deborah Davidson, Andrew Newman, Peter Thomson, Chris Darby, Ronny van Aerle

**Affiliations:** 1 Centre for Environment, Fisheries and Aquaculture Science (Cefas), Lowestoft, Suffolk, UK; 2 Argos Froyanes Ltd, GX11 1AA Gibraltar, Gibraltar; 3 Collaborative Centre for Sustainable Use of the Seas, University of East Anglia, Norwich, UK; 4 Centre for Sustainable Aquaculture Futures , University of Exeter, Exeter, UK

**Keywords:** *Dissostichus eleginoides*, Nototheniidae, Illumina sequencing, PacBio sequencing, Anti-freeze glycoprotein

## Abstract

**Background:**

Patagonian toothfish (*Dissostichus eleginoides*) is an economically and ecologically important fish species in the family Nototheniidae. Juveniles occupy progressively deeper waters as they mature and grow, and adults have been caught as deep as 2500 m, living on or in just above the southern shelves and slopes around the sub-Antarctic islands of the Southern Ocean. As apex predators, they are a key part of the food web, feeding on a variety of prey, including krill, squid, and other fish. Despite its importance, genomic sequence data, which could be used for more accurate dating of the divergence between Patagonian and Antarctic toothfish, or establish whether it shares adaptations to temperature with fish living in more polar or equatorial climes, has so far been limited.

**Results:**

A high-quality *D. eleginoides* genome was generated using a combination of Illumina, PacBio and Omni-C sequencing technologies. To aid the genome annotation, the transcriptome derived from a variety of toothfish tissues was also generated using both short and long read sequencing methods. The final genome assembly was 797.8 Mb with a N50 scaffold length of 3.5 Mb. Approximately 31.7% of the genome consisted of repetitive elements. A total of 35,543 putative protein-coding regions were identified, of which 50% have been functionally annotated. Transcriptomics analysis showed that approximately 64% of the predicted genes (22,617 genes) were found to be expressed in the tissues sampled. Comparative genomics analysis revealed that the anti-freeze glycoprotein (AFGP) locus of *D. eleginoides* does not contain any AFGP proteins compared to the same locus in the Antarctic toothfish (*Dissostichus mawsoni*). This is in agreement with previously published results looking at hybridization signals and confirms that Patagonian toothfish do not possess AFGP coding sequences in their genome.

**Conclusions:**

We have assembled and annotated the Patagonian toothfish genome, which will provide a valuable genetic resource for ecological and evolutionary studies on this and other closely related species.

**Supplementary Information:**

The online version contains supplementary material available at 10.1186/s12864-024-10141-4.

## Background

Patagonian toothfish (*Dissostichus eleginoides*) are found around the sub-Antarctic islands of the Southern Ocean. Larvae and juveniles occupy relatively shallow areas for the first few years of life, before migrating deeper, with adults having been caught as deep as 2500 m, living on or just above the shelves and slopes of the Southern Ocean [1, 2]. Patagonian toothfish and its closely related sister species, the Antarctic toothfish (*Dissostichus mawsoni*), belongs to a single genus which falls within the Nototheniidae family and, with a few exceptions around sub-Antarctic islands, their geographical distributions do not appear to overlap [3, 4]. Patagonian and Antarctic toothfish are also sustainably fished species and support valuable fisheries throughout the Sub-Antarctic regions [5–7], with many toothfish stocks being managed under or in line with the Convention for the Conservation of Antarctic Marine Living Resources (CCAMLR).

The two toothfish lineages have historically separated, with the Patagonian toothfish adapting to more temperate climates and Antarctic toothfish retaining all the genetic adaptations required to survive in the cold Antarctic waters [8]. This is reflected in physiological differences between the two species whereby in contrast to the Antarctic toothfish, no evidence has been found for the presence of anti-freeze glycoprotein (AFGP) in the blood of the Patagonian toothfish [9] or within its genome [10]. Other, more subtle adaptations of antarctic notothenioids to the Antarctic environment which could potentially be found within the genome of one or both species of toothfish include changes in membrane composition and the structure of protein translocation channels [11, 12], in the regulation of molecular chaperones [13–17], in the expression of haemoglobin and regulation of the circadian rhythm [12, 18–20], as well as in the structure of microtubules in the cytoplasm [21], though more work is required to obtain a complete picture of all the different ways in which this group has adapted to such cold conditions (see [22] for a recent review).

Recent advances in sequencing technologies have facilitated the sequencing of non-model species in a relatively cost-effective way. Availability of sequence data facilitates studies on many aspects of notothenioid fish biology, including observations that oxygen rich cold environments may have led to loss or reduction in haemoglobin expression [12, 19], the polar light environment has allowed the loss of some genes crucial in regulating circadian homeostasis [18], lower temperature variation has relaxed selective pressure which would otherwise have prevented the loss of the heat-shock response [15] as well as the increased expansion and expression of AFGP genes which prevent freezing of blood and tissues in the subzero temperatures of the antarctic [19, 23]. The Antarctic toothfish genome was sequenced in 2019 [24] and more recently, the genomes of 24 different notothenioids were also sequenced and assembled [19]. Importantly, whilst regions which carry important genetic signatures of selection have been characterised in the Antarctic toothfish genome, such as, for example, those controlling expression of haemoglobin and AFGP as well as regulation of the circadian rhythm and the heat-stock response [19, 24, 25], this has not been done in Patagonian toothfish. The genome of the Patagonian toothfish has not been entirely sequenced to date, and very limited genomic sequence information is available in public databases for this species. This lack of sequence information restricts studies on physiology and disease resistance, or on population genetics and comparative genomics analysis with other (related) fish species.

Given the important ecological role Patagonian toothfish appear to play in the Southern Ocean ecosystem as opportunistic carnivores or scavengers [2], the aim of this study is to address the lack of genome sequencing information by generating a high-quality genome assembly using a combination of long read (PacBio), short read (Illumina), and Omni-C sequencing data. Furthermore, the genome sequence was characterised and screened for the presence of the AFGP locus and subjected to phylogenetic analysis to identify any remaining evidence that AFGP genes may once have been present, and were subsequently lost following divergence from the last common ancestor shared with Antarctic toothfish [26]. The annotated Patagonian toothfish genome generated in this study will provide a valuable genetic resource for studying the evolution of adaptation of fish to cold waters, as well as other evolutionary and ecological studies on this species.

## Results

### Assembly and scaffolding of the genome

Long read sequencing using 6 SMRT cells generated a total of 77.1 gigabases (Gb), with an average of 12.8 Gb per SMRT cell. The longest read generated was 130 kb, with N90 and N50 values being 10 and 32 kb respectively. Illumina sequencing of the same DNA sample produced approximately 979 million read pairs (148 Gbp).

**Fig. 1:**
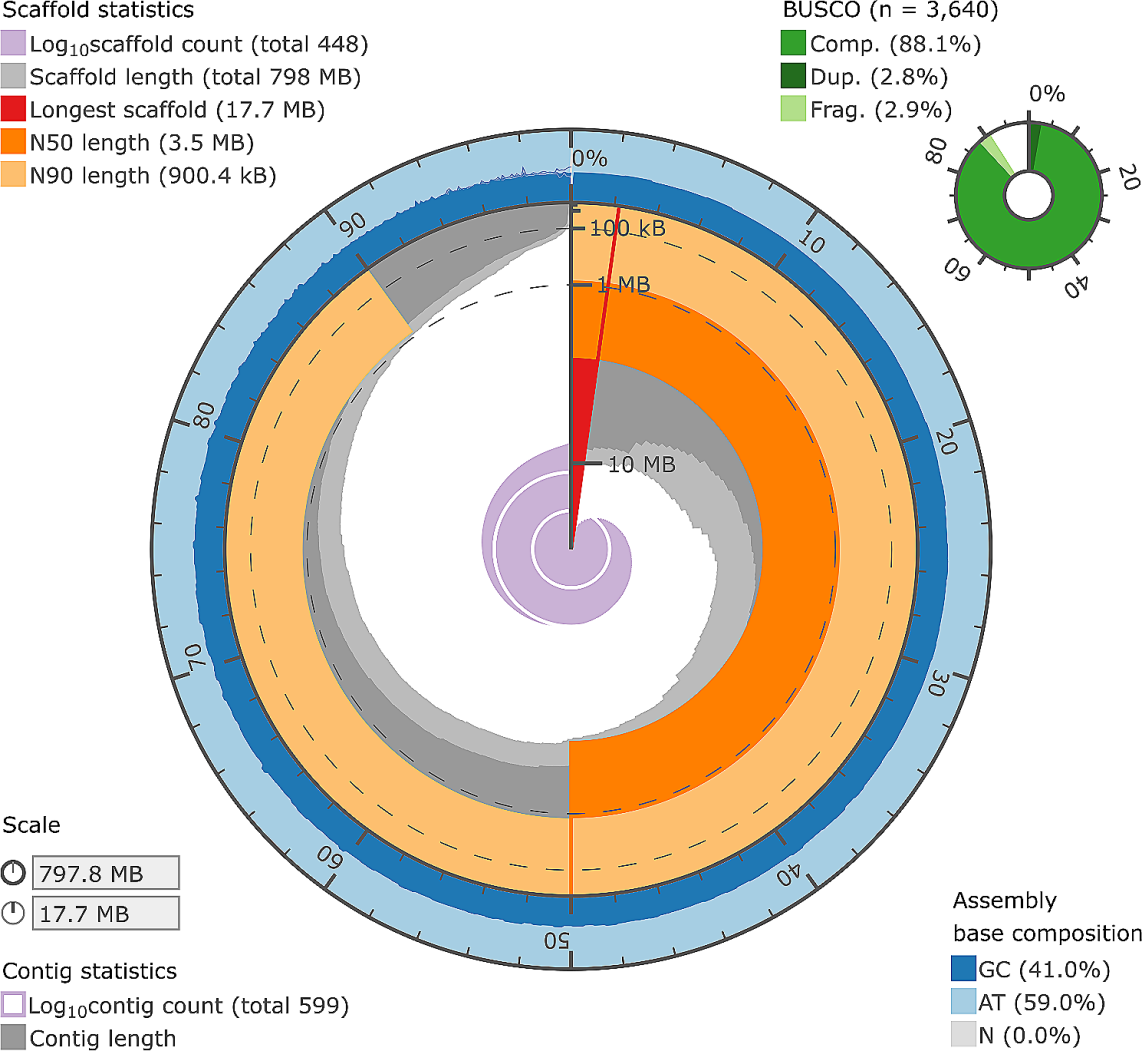
Visualisation of the Patagonian toothfish (*Dissostichus eleginoides*) genome assembly statistics. Red, dark, and light orange represent the longest, N50 and N90 scaffold lengths respectively, whilst the dark grey colour represents the length of each scaffold plotted against the cumulative length of all scaffolds on a circular axis, with the longest scaffold plotted nearest the red marker and scaffolds being sorted according to length. The light grey colour follows the same approach, but for contigs, rather than scaffolds. The outermost layer, plotted in blue, shows the GC content across the genome. The BUSCO score was determined by running BUSCO against the final set of gene annotations using ‘proteins’ mode and the OrthoDB v10 *Actinopterygii* database

Based on the Illumina sequencing library and a k-mer based statistical approach, the genome size was estimated to be approximately 762 Mb, with 242 Mb of repeats. The initial genome assembly, after polishing with Illumina reads and purging haplotigs from the assembly was 799 Mb, consisting of 593 contigs and with an N50 value of 2.54 Mb. Following assembly of the genome, the sequences derived from the Omni-C library were mapped to the assembled contigs. Table 1 shows a summary of the proportion of mapped and unmapped reads, the number of PCR duplicates as well as alignment results sorted into *cis* and *trans* pairs and estimated insert lengths for *cis* pairs. Initial results suggested a high proportion of unmapped or partially mapped read pairs in the library and relatively short distance interactions in those cases where both reads in a pair mapped to the same contig, suggesting the library may have had some contamination, there was insufficient DNA, or DNA was of insufficient quality prior to starting the Omni-C protocol. Despite this, when using the results to scaffold the genome with SALSA, the number of contigs in the genome assembly was reduced from 593 to 455, with the median contig length increasing from 2.54 to 3.55 Mb. Following scaffolding of the assembly, it was purged of any remaining haplotigs using stringent coverage thresholds, resulting in a final genome assembly of 448 contigs totalling 797.8 Mb (Table 1; Fig. 1) of which 253 Mb (31.76%) consisted of repeat elements (see Supplemental Table 2). Overall, including haplotigs, a k-mer based analysis of genome completeness using Merqury suggested the assembly was 97.62% complete. As part of the same analysis, the consensus quality value (QV) of the assembled, scaffolded, purged assembly was calculated as 42.09 (see Table 2).

**Table 1:** Mapping statistics (a) and classification (b) of the reads of the Omni-C library

**a**		**Number of reads**	**Percentage of reads**
	Paired reads	329,516,656	100.0
	Unmapped read pairs	55,047,299	16.7
	Mapped, unpaired reads	124,028,116	37.6
	Mapped, paired reads with PCR duplication	85,290,350	25.9
	Mapped, paired reads with no PCR duplication	65,150,841	19.8
**b**		**Number of reads**	**Percentage of mapped paired reads (no PCR duplication)**
	*Cis* Read Pairs	18,793,875	28.9
	*Trans* Read Pairs	46,356,966	71.2
	Valid Read Pairs (*cis* ≥ 1 kb + *trans*)	48,308,460	74.2
	*Cis* Read Pairs < 1 kb	16,842,381	25.9
	*Cis* Read Pairs ≥ 1 kb	1,951,494	3.0
	*Cis* Read Pairs ≥ 10 kb	1,420,337	2.2

**Table 2:** Statistics summarising the contiguity, quality, and completeness of the Patagonian toothfish (*Dissostichus eleginoides*) genome assembly

	Primary Contigs	Haplotigs	Overall
Total Size (Mb)	798	651	1449
Scaffold N50 Length (kb)	3550	201	1247
Scaffold N90 Length (kb)	900	103	125
Number of Contigs	448	3524	3972
QV score	42.09	37.99	39.78
Completeness score	92.51	68.31	97.62

### Transcript assembly, analysis, and genome annotation

RepeatMasker identified 253.40 Mb of repeats which were soft masked prior to genome annotation and used by EVidence Modeler (EVM) whilst annotating genes. Augustus, SNAP and GlimmerHMM were used to carry out ab initio gene prediction, with all three using BUSCO predicted genes as training data, but with Augustus also using results from the mapping of reference proteomes and RNA sequencing data to the genome as *‘hints’*. In total there were 35,543 predicted protein-coding sequences after combining the various predictions using EVM, as well as 6887 predicted tRNA sequences. Predicted protein coding regions in the final set of gene annotations included complete copies of more than 90% of the proteins present within the OrthoDB v10 *Actinopterygii* database, according to BUSCO. Functional annotation of the genome included adding 51,105 gene ontology terms, 4438 signal peptide predictions and 7453 transmembrane annotations.

**Fig. 2:**
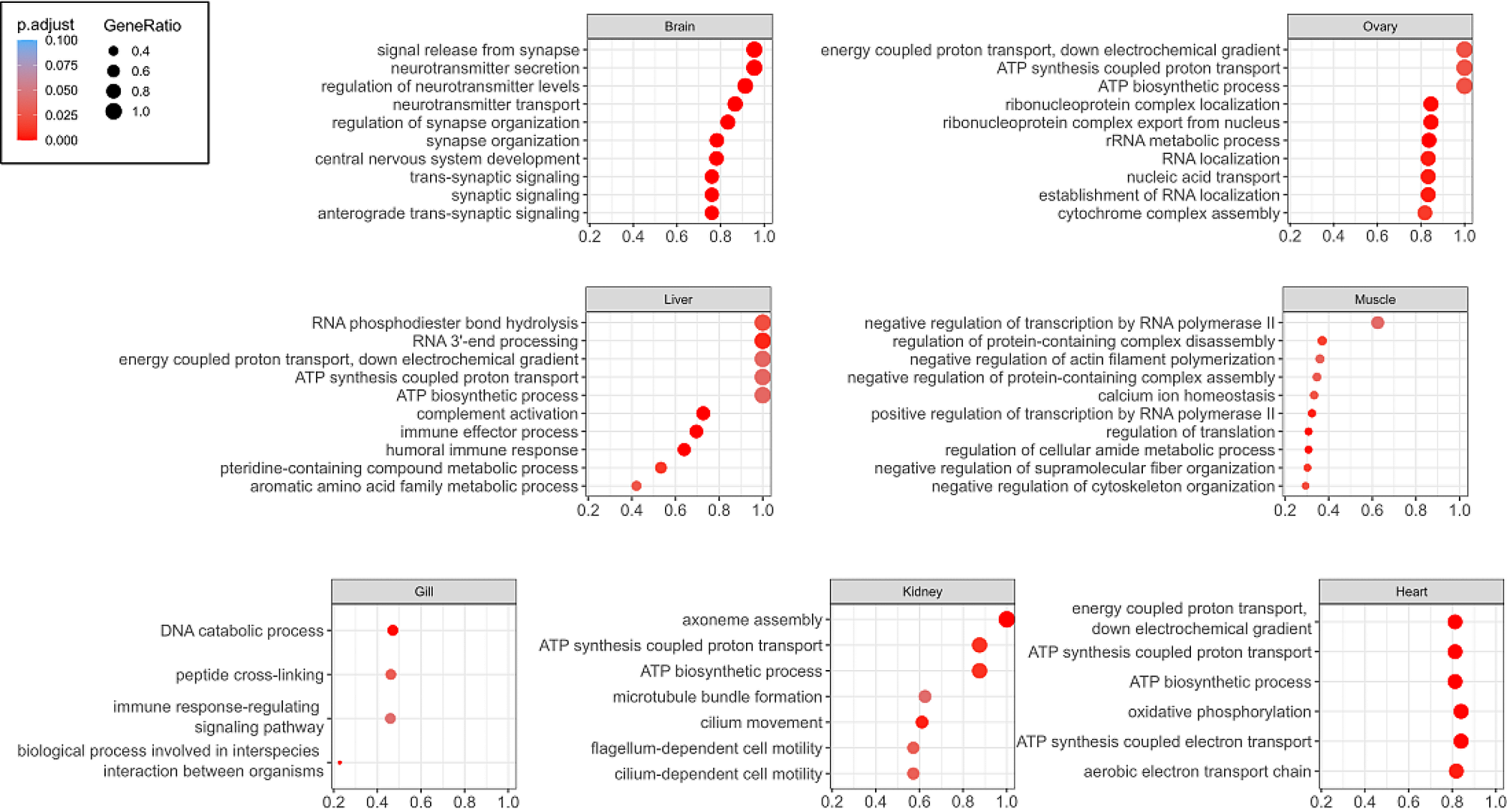
Dot plots showing enriched gene ontologies as identified by Gene Set Enrichment Analysis (GSEA) in each tissue. The Top 10 (or all) Gene Ontologies (Biological Processes) are shown. The size of the solid circles is proportionate to the number of genes represented in the corresponding category and the colour indicates the significance value

A total of 22,617 different genes were expressed in at least one of the tissues, of which 3762 genes were expressed in every tissue (Table 3). Each tissue had uniquely expressed genes, with the brain (*n* = 358) and ovary (*n* = 123) expressing the most, whilst the spleen did not express any unique genes. The top 10 biological processes that were found to be enriched in each of the tissues are shown in Fig. 2 (see Supplemental Table 3 for complete lists of enriched gene ontology terms and corresponding expressed genes for each tissue). As expected, the processes identified to be enriched in each of the tissues related to the specific function(s) of those tissues. For example, the brain, which had the highest number of uniquely expressed transcripts, was enriched for biological processes relating to signalling across synapses, neurotransmitter secretion and central nervous system development. Some tissues, such as the intestine, did not have any enriched biological processes, whilst the spleen only had one, which was *‘protein localisation to the endoplasmic reticulum’*. The small number of enriched terms for the spleen could possibly be due to overlapping functions with other tissues.

**Table 3:** The number of transcripts (total and unique) expressed in each tissue sample

Tissue Sample	Number of transcripts expressed	Number of uniquely expressed transcripts
Brain	17,259	358
Gill	14,626	62
Ovary	12,048	123
Heart	12,179	11
Intestine	15,203	20
Kidney	14,027	10
Liver	5148	18
Muscle	10,683	18
Spleen	11,493	0
Overall	22,617	620

### Phylogenetic analysis

**Fig. 3:**
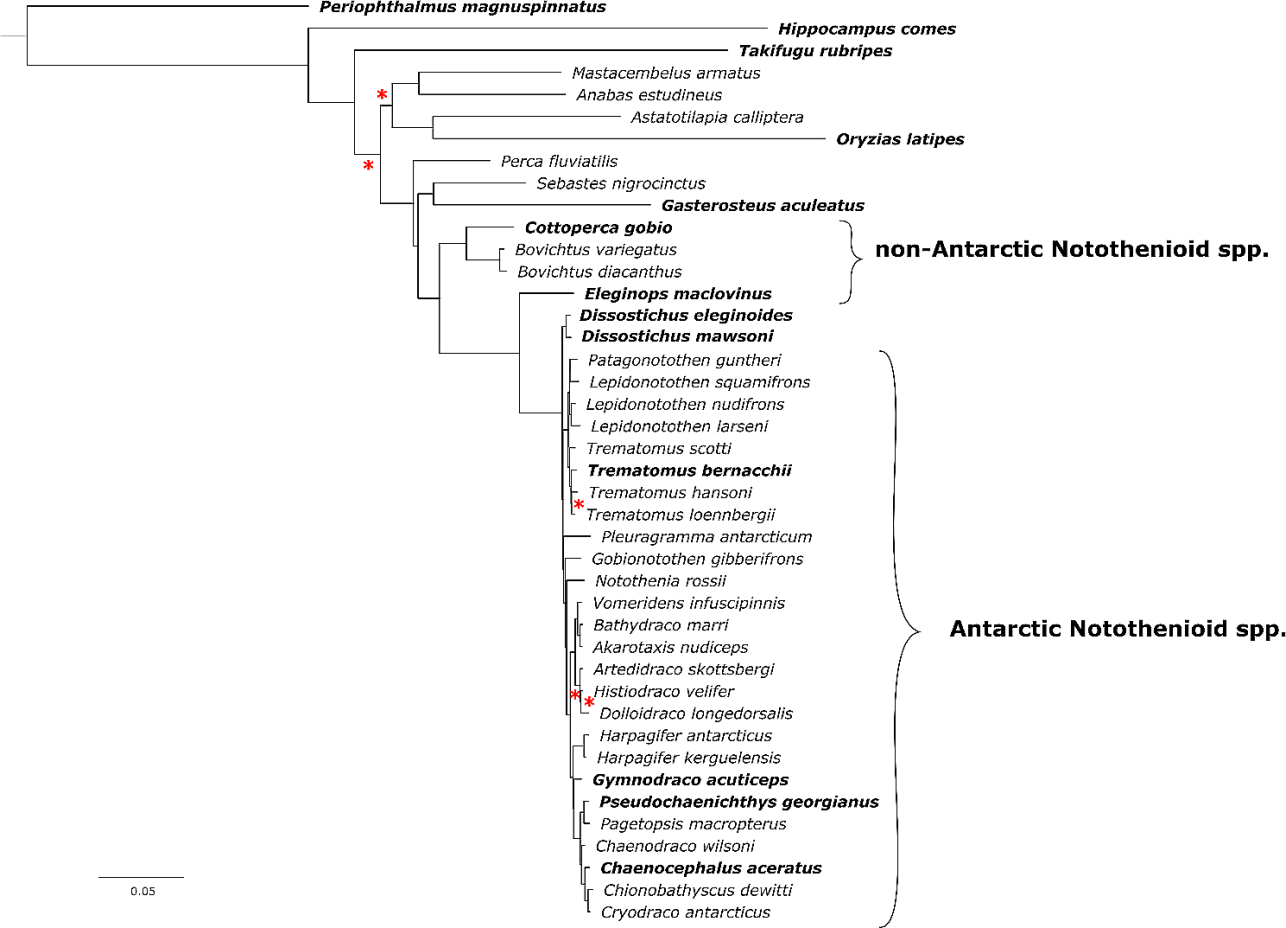
Maximum likelihood phylogenetic analysis of 42 percomorph fish species, including 32 species of notothenioids and 10 outgroups. Equal branch lengths, but different rates of evolution, were used for each one of 151 initial nucleotide partitions. A relaxed hierarchical clustering algorithm was used to examine the top 10% of partition merging schemes and identify the best model. An ultrafast bootstrapping approach was used with 1,000 replicates. Any clades with less than 95% support are marked with a red asterisk. Species for which gene annotations were available are highlighted in bold

Bootstrap analyses provided high confidence for the maximum likelihood phylogenetic tree topology generated based on an alignment of 220 kb of DNA sequences from 151 orthologs and 42 percomorph fish species (Fig. 3). There were, however, a few exceptions, including locations within some of the deeper branches of the tree, outside of the notothenioids clade, as well as for some *Trematomus* and Artedidraconidae species. In the latter case, poor support for placement of individual species within these taxonomic groups did not seem to impact overall placement of the *Trematomus* genus or the Artedidraconidae family within the phylogeny tree. The Patagonian toothfish sequences branched with the Antarctic toothfish near the root of the Antarctic notothenioid clade.

### Assembly of the mitochondrial genome

**Fig. 4:**
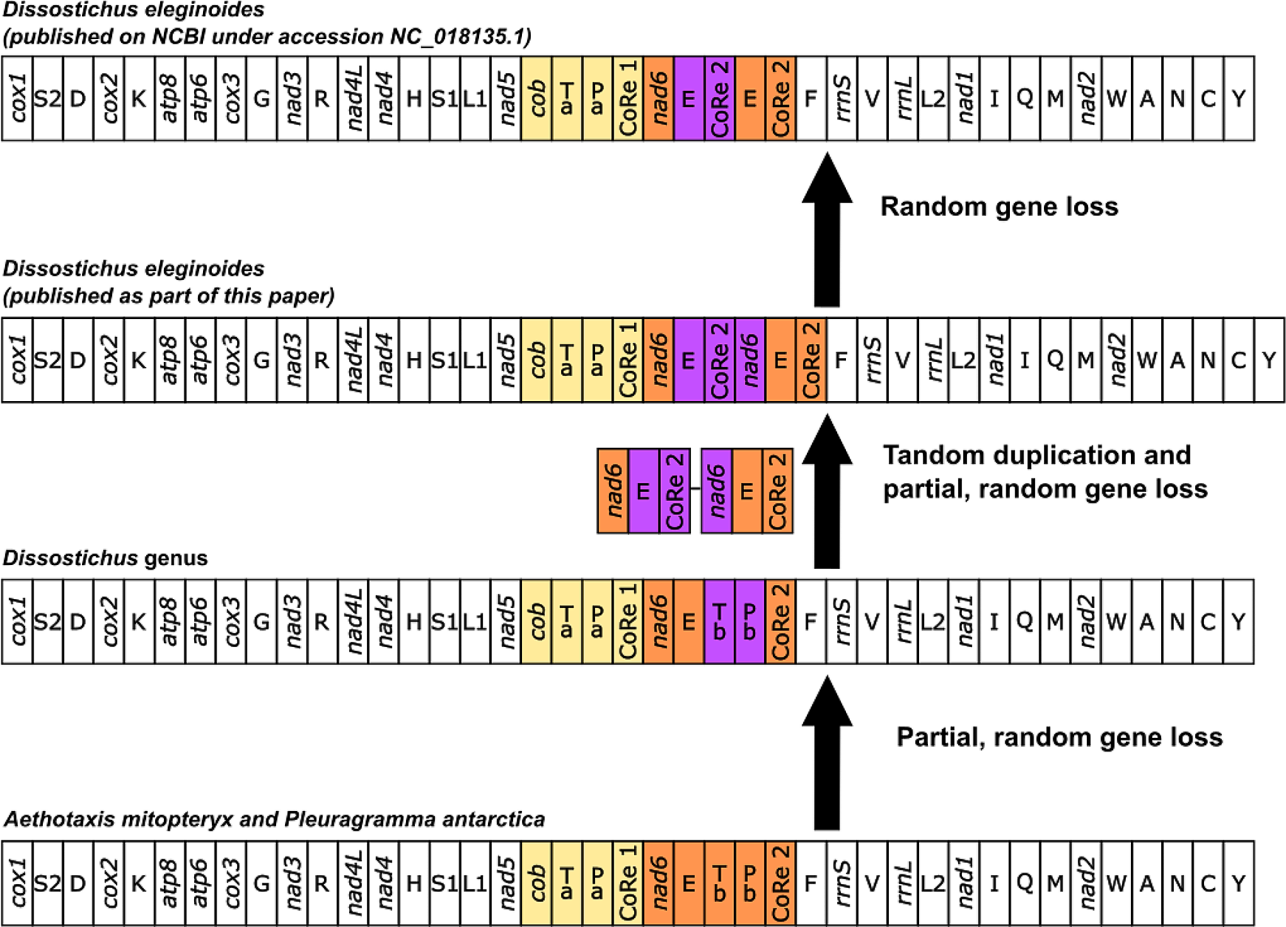
An updated proposal for mitochondrial gene order evolution based on existing work by Papettiet al. [24]. Gene order is linearised starting from *cox1*. Genes transposed/duplicated with respect to the gene order expected for a mitochondrial genome from a *‘standard’* vertebrate organism are shown in a yellow (gene belonging to the 5’ duplicated block) and orange (gene belonging to the 3’ duplicated block) background. Copies of the genes partially lost during the genomic rearrangement are framed in purple. Nomenclature: *atp6* and *atp8*, ATP synthase subunits 6 and 8; *cob*, apocytochrome b; *cox1-3*, cytochrome c oxidase subunits 1–3; *nad1-6* and *nad4L*, NADH dehydrogenase subunits 1–6 and 4 L; *rrnS* and *rrnL*, small and large subunit ribosomal RNA (rRNA) genes; X, transfer RNA (tRNA) genes, where X is the one-letter abbreviation of the corresponding amino acid; CoRe, Control Region

Assembly of the mitochondrial genome using a combination of PacBio and Illumina sequences resulted in a circular genome of 19,459 bp in length. Several duplicated genes were found, which included various deleterious mutations. The gene order in the Patagonian toothfish mitochondrial genome was different in comparison to other vertebrate species, as was previously described by Papetti et al. [27].. However, the genome we assembled had an additional pseudogene for *nad6* which was not present in the previously published Patagonian toothfish mitochondrial genome (see Fig. 4 and NC_018135.1).

### Comparative genetics

Species highlighted in bold in the phylogenetic tree were used to identify orthologus genes, including both species within (*n* = 9) and outside (*n* = 5) of the notehenioid clade (see Fig. 3 and Supplementary Table 1). The *Notothenia* genus was included in both the phylogenetic and orthology analysis, but limited availability of gene annotations meant that unfortunately different species ended up being included in each analysis (see Supplementary Table 1). Overall, 27,297 orthogroups were identified, of which there were between 17,000 and 21,000 orthogroups found per species and a set of 8442 core genes shared across every species. Most of the species included in the analysis had over 94% of genes assigned to a specific orthogroup, with only *Chaenocephalus aceratus*, *D. eleginoides* and *Eleginops maclovinus* having a lower number of genes identified as orthologs (80–85%). Whether differences in the latter were due to a different approach to gene annotation, or to gaps in taxonomic coverage leading to less sensitive identification of orthogroups within this species, or the species adapting to a different evolutionary niche, requires further investigation.

### Analysis of the antifreeze glycoprotein locus

Alignment of RNA reads against each transcript predicted for haplotype 1 of the antifreeze glycoprotein (AFGP) locus (see HQ447059.1) resulted in complete coverage for trypsinogen, trypsinogen-like proteases and translocate gene coding transcripts in multiple tissues. Approximately 20–30% coverage was observed for transcripts coding for the chimeric antifreeze glycoprotein/trypsinogen-like protease. However such coverage was only observed for regions of the gene sharing homology with the trypsinogen-like protease gene, and not for the part of the transcript conferring the antifreeze phenotype. None of the RNA reads aligned against any of the transcripts coding for the AFGP genes.

Two candidate AFGP loci were identified, one from the primary assembly, and another which had previously been identified as a haplotig. These candidate loci were the only ones aligning against the AFGP haplotypes published for *D. mawsoni* (see HQ447059.1 and HQ447060.1, > 1000 bp and > 90% identity). The candidate locus from the primary assembly was also the only one to include orthologs for the *hsl* and *tomm40* genes which were found in different notothenioids, and where the corresponding genome regions included other features associated with the AFGP locus, such as protease and trypsin genes, as well as AFGP genes, in the *Pseudochaenichthys georgianus* genome.

To identify misassemblies, PacBio sequencing reads were aligned against the two candidate loci for *D. eleginoides*, which showed that each of the loci had between 33 and 39x coverage, with mean mapping quality > 55. Manual inspection of the alignment results showed many reads which mapped across almost the whole length of the AFGP loci, with no obvious signs of misassembly, insertions or deletions.

**Fig. 5:**
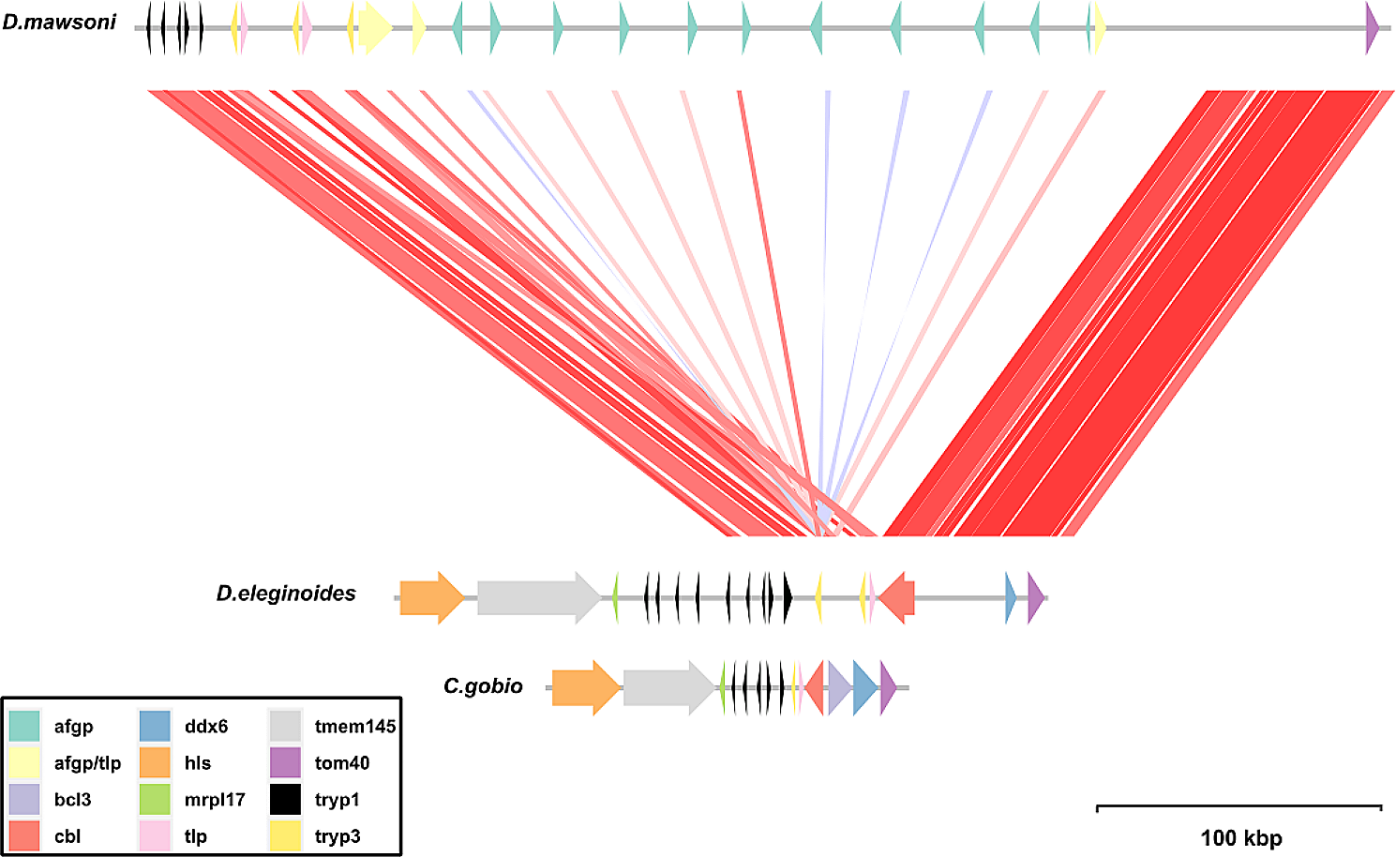
A map of the antifreeze glycoprotein (AFGP) locus for *Dissostichus eleginoides*, *Cottoperca gobio*, and *Dissostichus mawsoni*. Nucleotide BLAST alignments between Patagonian and Antarctic toothfish with more than 90% identity and a score of greater than 2830 are shown in colours ranging from red (90%) to blue (100%), which represent alignments with a high and low percentage identity. Arrow colours correspond to conserved genes; arrow heads indicate gene orientation

Figure 5 shows a schematic represtentation of the published AFGP loci from *D. eleginoides*, *D. mawsoni* and *Cottoperca gobio*, indicating regions of high similarity across the toothfish loci. The *tryp3*, *tryp1* and *tlp* genes appear to have been duplicated in the *Dissostichus* genus relative to *C. gobio*. The *ddx6*, *tmen145* and *cbl* genes are not consistently observed across every species, though there could possibly be variation in the level of completeness of gene annotation in each of the three species. The most notable difference between the two species of *Dissostichus* is the complete absence of any AFGP gene within the AFGP locus for *D. eleginoides*. There is, however, a region found in each copy of the AFGP gene from *D. mawsoni* that shares some homology with a *tlp* gene from *D. eleginoides*. The *tlp* and *cbl* genes appeared to be functional in the *D. eleginoides* genome, based on RNA sequencing data, were present in the same order and orientation, and situated roughly the same distance from each other as in *C. gobio*. In contrast, the tlp and *cbl* genes were separated by a dozen or more tandem repeats of the AFGP gene in *D. mawsoni*.

## Discussion

### Genome assembly and quality assessment

There are many criteria for evaluating the quality of a genome, with the Vertebrate Genomes Project (VGP) having recently defined several metrics designed to assess continuity, base pair accuracy, functional completeness and chromosome status [28]. The genome for *D. eleginoides* presented here is 797.8 Mb in size and has a base pair quality of > 40, k-mer and BUSCO completeness scores > 90%, and an N50 value of over 1 Mb. We also generated extensive RNA sequencing data from multiple tissues using both short and long reads, which can be used to more effectively annotate genes, identify splice variants and confirm predictions made using *de novo* prediction algorithms [29–31]. Our genome assembly measures well when compared against a range of quality criteria set by the VGP, but one limitation is that the Omni-C protocol was not sufficient to achieve chromosomal level scaffolding nor haplotype phasing. This would have been facilitated by combining the latest assembly algorithms with a newer generation of long read sequencing technology such as PacBio HiFi reads [32]. Depending on gene density, assemblies with a minimum N50 value between 0.2 and 1 Mb have been shown to be sufficient to yield consistent results when being used for synteny analysis [33]. This suggests that contiguity in our genome assembly is high enough to support investigation of the evolution of long, repetitive loci, like the AFGP locus [25], or the consequences of having different karyotypes in the two otherwise closely related species of toothfish [34].

In this study, we conducted several cross-species comparisons including a phylogenetic analysis based on a large number of orthologous, single copy genes from the nuclear genome, an updated examination of unusual variations in the order of mitochondrial genes observed within the *Dissostichus* genus [27], and a comparison of the order and number of genes found within the AFGP locus [19]. These comparisons were facilitated by highly contiguous assemblies with good base pair accuracy and gene annotations. However, the quality of genome assemblies varied across different notothenioids (Fig. 3). Only 6 out of 32 genomes submitted to various sequencing repositories included published gene annotations, making phylogenetic analysis, identification of orthologs, and other comparisons across species challenging. In addition to missing annotations, previous studies have reported variation in the level of completeness calculated for the genome assemblies of different notothenioids. For example, gene annotations provided for the *N. corriiceps*, *C. aceratus*, *E. maclovinus*, *D. mawsoni* assemblies had BUSCO completeness scores ranging between 80 and 97% [12]. Another related issue is the level of contiguity of assemblies, with the first genome published for Antarctic toothfish [24] having much smaller scaffold lengths than the Patagonian toothfish (e.g. N90 values of 202.7 kb vs. 900.4 kb, respectively), despite having a similar estimated genome size. As sequencing chemistry, library preparation and assembly methods continue to improve and become standardised, it will allow the production of higher quality assemblies, and streamlined comparisons across species.

### The Patagonian toothfish genome

Within the existing literature, phylogenetic analysis of the notothenioids has been carried out using a range of techniques, including RAD-Seq [35, 36], small sets of nuclear or mitochondrial markers [8, 37–40], as well as those based on a more comprehensive set of nuclear markers [19]. Balushkin et al. [41] proposed a single clade based on morphological criteria, which included *Pleurogramma antarcticum*, two species of *Dissostichus*, *Aethotaxis mitopteryx*, and *Gvozdarus svetovidovi*. However, recent studies using genetic data have often identified this group as being paraphyletic [19], with *P. antarcticum* as an outgroup, but overall there has been insufficient evidence to reject the monophyletic hypothesis [35] or the proposed lineages were supported by weak bootstrap values [8, 39]. Adding to the uncertainty, none of the analyses based on molecular evidence appear to include all of the species within the proposed group, with *A. mitopteryx* and *G. svetovidovi*, among others, often not being included in the analyses. Comprehensive phylogenetic analyses using genome-wide sequence information, e.g. using a large number of gene orthologs (this study), nuclear markers [19] or SNPs [35], suggest that these species are very close to the root of the Antarctic notothenioids clade, though which species is closest to the base (*P. antarcticum* [35], *Dissostichus* spp [19]. or another species from the same group) still is subject to some level of disagreement and merits further investigation.

Analysis of the transcriptome and genome of Patagonian toothfish confirmed earlier work which found no evidence for the presence of AFGP genes within the genome [10], nor expression of proteins within the blood [9]. Notothenioids lacking the antifreeze glycoprotein phenotype appear to either express AFGP but at very low levels and with mutations in key amino acid motifs [26], or they do not express AFGP and lack the AFGP locus in their genomes (*e.g. Patagonotothen tessellata*, *P. ramsayi*, and *D. eleginoides* [10, 26]). The absence of AFGP within Patagonian toothfish suggests that either the species diverged prior to acquisition of the AFGP genotype within the notothenioids, or the gene became degraded and was subjected to large-scale mutations, or it was lost after the species occupied ecological niches outside of the colder waters of the Antarctic. The data generated in our study allowed for a much more detailed analysis of gene content within the AFGP locus than was possible with earlier work based on Southern blot analysis, and provided no evidence for any degraded or mutated form of AFGP genes within the Patagonian toothfish genome. Unlike degeneration or mutation of the AFGP genes, it is not possible to rule out the possibility that AFGP genes were present within a common ancestor of Patagonian and Antarctic toothfish and subsequently lost. In contemporary notothenioids, a high number of copies of the gene seems to be required to survive colder habitats [10, 19], as is observed for Antarctic toothfish, for example. It is plausible that lower levels of expression of AFGP, and therefore fewer copies of the AFGP gene, would have been required for fish exploiting a slightly warmer, but still cold Southern Ocean of the recent geological past. The geological evidence suggests a gradual cooling of the climate over millions of years, with glaciers first forming in Antarctica around 35 Ma [42, 43], temperatures in the Southern Ocean falling another 6–7 °C around 14 Ma [44] with signs of more recent cooling within the last few million years [19]. More recent phylogenetic analyses suggest the Patagonian toothfish diverged from other notothenioids relatively early on, when temperatures in the Antarctic were probably warmer than they are at present, leaving open the possibility that its AFGP locus could indicate an earlier state, prior to large scale duplication of the AFGP gene which subsequently led to the high expression of AFGP seen in other species. Reconstructing the evolution of genes within this locus with any degree of confidence is likely to require analysis of more examples from members of the Pleuragrammatinae subfamily, as well as other species thought to be lacking copies of the AFGP gene.

The mitochondrial genome had an additional pseudogene for *nad6* which was not present in a previously published genome (see Fig. 4 and NC_018135.1). One possible interpretation of these results is there is more than one haplotype present within Patagonian toothfish populations, which may be more likely in notothenioid fish than would be expected based on observations in other vertebrates, given the recent observations of heteroplasmy involving copy number variations in the nad6 and control regions of the mitochondrial genome of a couple of different species of icefish [45]. However, discrepancies between different mitochondrial genomes published for notothenioids have been noted before when using different sequencing and library preparation techniques, such as species within the *Trematomus* genus [45, 46]. These types of discrepancies could potentially occur when using short read sequencing data to assemble the mitochondrial genome, a challenge which appears to be more difficult for Antarctic notothenioids due to the standard vertebrate gene order not being conserved, with multiple events, such as tandem duplication, inversion and partial gene loss having been proposed to explain the gene order present in published genomes [27]. Therefore, the mitochondrial genome published in this paper is a useful contribution to those studying unique changes in the gene order of mitochondria for Antarctic notothenioids since it is based on long read sequencing data, and therefore less likely to be misassembled.

### Practical applications

Patagonian toothfish support fisheries around the sub-Antarctic regions and understanding spatial stock structure is an important component in precautionary and sustainable management. Multiple studies have been carried out to identify stock structure of both toothfish species, including analysis of tagged fish movements [4, 47], mineral deposits within otoliths [48], microsatellites [49], and SNP/RAD-Seq phylogenetic markers [50]. The Patagonian toothfish genome sequences provided by this study will further strengthen the resources available for population genetics, allowing identification of the most suitable restriction enzymes to use for RAD-Seq analysis [51, 52], guiding the choice of neutral markers or restriction enzymes based on proximity to gene coding regions [53] and allowing a scan of the genome to identify regions showing higher levels of adaptive or balancing selection [54, 55]. In short, sequencing the genome makes it easier to ensure any markers used to study population structure are neutral, frequent, and widely dispersed within the genome, thereby reducing bias.

Climate change is expected to have significant impacts on antarctic notothenioids, which are adapted to life in the cold, stable waters of the Southern Ocean. Changes in water temperatures could affect growth rate, metabolism, and reproductive success of these fish species, with loss or reduction in expression of haemoglobin [12, 18, 19] and impaired heat shock response [13–17] being specific examples of how warmer conditions could be problematic. Additionally, there is the potential for a more general disturbance in the abundance and distribution of their primary food resources. Furthermore, non-native fish species, including those that do not possess anti-freeze glycoproteins and were restricted from living in the colder waters, could potentially expand into notothenioid habitats, possibly competing with and preying on native notothenioids in the antarctic. The genomic resources developed in our study can be used to provide valuable insights into how antarctic notothenioids will respond to climate change and the potential impacts on their populations and ecosystems. By combining genomic data with ecological and environmental data, a more comprehensive understanding of how these fish are adapting to a rapidly changing world can be developed.

## Conclusion

In this study, we produced a high-quality genome assembly for the Patagonian toothfish, an ecologically and economically important fish in the sub-Antarctic regions of the Southern Ocean. Predicted gene sequences, together with the transcriptomic data generated for a variety of tissues in this study, will facilitate studies on physiology, disease, reproduction, and population genetics in this species. Our work found no evidence of the presence of AFGP genes in the Patagonian toothfish genome. Phylogenetic analysis based on a set of orthologous protein sequences showed that the Patagonian toothfish is near the root of the Antarctic notothenioids clade. The genome will provide a valuable genetic resource for physiological, ecological, and evolutionary studies on this species.

## Methods

### Sample collection

A Patagonian toothfish (*D. eleginoides*) mature female (length = 95 cm, weight = 8.18 kg) was caught at a depth of approximately 1300 m, in the fishing area CCAMLR subarea 48.3B (South Georgia). Spleen, muscle, liver, kidney, intestine, heart, ovary, gills, and brain samples were collected and preserved in RNAlater™ (Invitrogen).

### DNA extraction

High molecular weight DNA was extracted from pooled visceral tissues (spleen, liver, and kidney) stored in RNAlater™ using the Qiagen Genomic tip 500G and the manufacturer’s recommended protocol. Briefly, 400 mg of toothfish visceral tissues were ground to a powder with liquid nitrogen in a pre-cooled mortar and pestle and then digested in 20 ml of G2 buffer containing RNase and proteinase K for 2 h at 50 °C. Following digestion, the sample was loaded onto a pre-equilibrated genomic tip 500G. The column was washed twice with 15 ml Buffer QC and eluted in 15 ml buffer QF. The DNA was precipitated using 0.7 volumes (10.5 ml) of isopropanol and centrifugation at 5000 g for 15 min at 4 °C; the pellet was washed with 4 ml of cold 70% ethanol and centrifuged at 5000 g for a further 10 min at 4 °C. The DNA pellet was air dried for 10 min and resuspended in 500 µl of TE buffer and stored at −80 °C.

### RNA extraction

RNA was extracted from the nine individual tissue samples stored in RNAlater™ using the Direct-zol™ RNA Miniprep Plus kit (Zymo Research) according to the manufacturer’s instructions. Briefly, 50 mg of each tissue were ground to a powder with liquid nitrogen in a precooled mortar and pestle and lysed in 600 µl of TRI Reagent. The lysed samples were transferred to clean 1.5 microtubes and equal volumes of absolute ethanol added. The samples were thoroughly mixed and 700 µl transferred to Zymo-Spin IIICG columns and centrifuged at 13,000 g for 30 s. The flowthroughs were discarded, and the columns treated with DNase I prior to washing with 400 µl of Direct-zol RNA prewash, followed by a wash with 700 µl of RNA wash buffer. The RNA samples were then eluted in 100 µl DNase/RNase free water and stored at −80 °C.

### Genome sequencing

Genomic DNA libraries for PacBio Sequel were generated and run on 6 SMRT cells at the Exeter Sequencing Facility (University of Exeter) using the SMRTbell Express Template Prep Kit 2.0, following the protocol described online [56]. An additional enzymatic digest step to remove any linear molecules was included, as described on page 13 of the protocol, before size-selection of > 20 kb fragments on a high pass cassette on the Blue Pippin (Sage Science, MA, USA).

The Illumina sequencing DNA library was prepared from the same DNA sample as for PacBio using the NEBNext® Ultra™ II FS DNA Library Prep Kit for Illumina (New England Biolabs). The Omni-C library was prepared from a separate visceral sample using the Dovetail™ Omni-C™ Kit with Library Module and Primer Set for Illumina (Dovetail Genomics, CA, USA) according to the manufacturer’s protocol. Both libraries were sequenced using an Illumina SP flow cell on an Illumina NovaSeq (2 × 150 bp protocol), following manufacturer’s instructions (Illumina, San Diego, CA, USA), at the Exeter Sequencing Facility (University of Exeter).

### RNA sequencing

RNA quality was assessed with an Agilent TapeStation using the RNA Analysis ScreenTape System (Agilent). Extracted RNA from brain, muscle, gills, and kidney passed the RIN score threshold required for IsoSeq sequencing. Equimolar amounts of RNA from these samples were pooled and used to generate a single SMRTBell library [57] using version 3 chemistry, with equal amounts of the library being run over 3 SMRT cells on the PacBio Sequel. For short read sequencing, mRNA libraries were prepared for all nine tissue samples using the TruSeq Stranded mRNA sample preparation kit (Illumina) and run on an Illumina NovaSeq (2 × 150 bp protocol). All the RNA libraries were sequenced at the Exeter Sequencing Facility (University of Exeter).

### Assembly and assessment of genome

Genome size was estimated by using Jellyfish 2.2.10 [58] to count kmers in the Illumina reads, followed by modelling of genome size using GenomeScope Release 1 [59]. The genome was assembled from the PacBio reads with Canu version 2.2 (-pacbio-raw and expected genome size 800 Mb) [60]. Illumina reads were aligned against the assembly using minimap 2.20-r1061 (default parameters) [61, 62], followed by polishing using Pilon 1.24 (parameters used: --fix-bases --diploid. Throughout the present study, Samtools 1.15 was used for sorting and/or indexing of alignment results [63].

PacBio reads were aligned against the assembly using 2.20-r1061 (default parameters and -I 1600G, --secondary = no), followed by removal of haplotigs using Purge_haplotigs 1.1.2 (default parameters and -l 10 -m 65 -h 150) [64].

Reads from the Omni-C library were mapped to the assembled primary contigs using bwa 0.7.17-r1188 [65], and filtered using pairtools 0.3.0 as described in the Omni-C protocol published online [66]. The BamToBed script (bedtools 2.30.0) and GNU sort were used to convert and sort the results, with SALSA 2.3 being used to carry out scaffolding (default parameters and -e DNASE -m yes) [67]. Following scaffolding any remaining haplotigs were purged, using the same approach as before, but with the ‘middle coverage’ threshold set to lower value of 62.

Quality and completeness of the assembly and genome was assessed with Merqury 1.3 and Meryl 1.3 (Illumina reads and a kmer-size = 21 bp) [68, 69]. Additionally, BUSCO 4.1.2 was used to compare predicted proteins or transcripts against v10 of the OrthoDB for species in the Actinopterygii lineage [70–72].

### Processing and mapping of IsoSeq3 and illumina RNA sequencing data

For the IsoSeq3 data, the ccs v4.2.0 software was used to output circular consensus sequences with a minimum predicted accuracy of 0.9. Barcodes were trimmed using lima v1.11.0 (--isoseq --peek-guess). Isoseq3 v3.3.0 refine command was used to trim poly-adenosine tails and remove concatemers. Full length non-chimeric (FLNC) reads were converted to fastq format using bam2fastq v1.3.0. Initial processing of IsoSeq3 data was undertaken with SMRT Link version 9.0.0 software [73] and minimap 2.20 (-u f -x splice:hq) was used to align FLNC reads against the draft genome [61, 62].

For each sample, Illumina paired end short reads sequencing data were separately aligned against the draft genome using hisat 2.2.1 (default options and -rna-strandness RF --downstream-transcriptome-assembly) [74].

StringTie 2.1.4 (with long read option for IsoSeq3 data and default parameters for Illumina data) was used to assemble reads into potential transcripts [75].

### Annotation of genome

RepeatModeler 2.0.1 was used to create a custom repeat library with the long terminal repeat (LTR) structural discovery pipeline enabled [76], together with the dependencies TRF 4.09 [77], RECON 1.08 (maximum sample size set to 81 Mb) [78], RepeatScout 1.0.6 [79] and LTR_Retriever 2.9.0 [80]. RepeatMasker 4.1.1 was then used to soft mask repeats in the assembly [81], with RMBlast 2.10.0 acting as a search engine [82] and minimum alignment score set to 250.

StringTie 2.1.4 was used to merge transcripts, with the stringtie2gff3 utility from the Funannotate 1.8.9 package being used to convert transcript coordinates into the GFF3 format [75]. Gene coding regions were predicted using Funannotate 1.8.9 and v10 of the OrthoDB (--organism other --max_intronlen 500,000 --repeats2evm --busco_db actinopterygii). Additional information used by Funannotate to train gene prediction algorithms included genes predicted using StringTie2 and proteins from the *Trematomus bernacchii* (NCBI Genbank Accession GCF_902827165.1), *Cottoperca gobio* (GCF_900634415.1), *Notothenia coriiceps* (GCF_000735185.1), and *Pseudochaenichthys georgianus* (GCF_902827115.1) reference genomes.

InterProScan 5.56-89.0 was used to carry out functional annotation (default analysis modules) [83, 84]. Functional annotation of gene coding regions were detected by Funannotate 1.8.9, incorporating InterPro and Gene Ontology (GO) terms based on InterProScan results, gene and product names based on a BlastP search of predicted proteins against UniProt DB 2022_01 [85, 86], as well as additional annotations from Pfam-A, MEROPS [87], CAZYme [88], BUSCO2 [71, 72], and Phobius analyses [89].

### Phylogenetic analysis

Species within (*n* = 22) and outside (*n* = 10) of the notothenioid clade were chosen for phylogenetic analysis (see Supplemental Table 1 for full list of species). BUSCO 4.1.2 was run in genome mode against genomes from each species [71, 72], with Augustus 3.3.3 used for prediction of gene coding regions [90], and v10 of the OrthoDB for species in the Actinopterygii lineage as a reference [70]. A custom python script was then run against BUSCO output to identify single copy orthologs consistently observed across every species, with the results being grouped by ortholog. MACSE 2.05 trimNonHomologusFragments function was used to trim non-homologous sequences and each orthologue aligned separately using MACSE 2.05 alignSequences function [91]. Translated amino acid sequences were checked by HmmCleaner 0.180750, and regions presumed to be sequencing error, rather than biological variation, were masked. MACSE 2.05 reportMaskAA2NT function was used to mask regions identified as problematic by HmmCleaner and to additionally process the aligned sequences (-min_NT_to_keep_seq 30 -min_seq_to_keep_site 4 -min_percent_NT_at_ends 0.9 -dist_isolate_AA 3 -min_homology_to_keep_seq 0.3). Trimmed, aligned, homologous sequences longer than 500 bp were available for 151 orthologs from 42 different species, making up a total of 220,443 bp of sequencing data.

Maximum likelihood analysis of the trimmed, aligned, homologous sequences longer than 500 was carried out using IQ-TREE 2.2.0.3 [92], with each partition sharing the same set of branch lengths, but allowing different rates of evolution, using the relaxed hierarchical clustering algorithm to examine the top 10% of partition merging schemes and identifying the best option [93], with ultrafast bootstrapping (1000 replicates) [94], and identifying the best-fit substitution model following identification of the best partitioning scheme [95] (-s allseqs.fas -p allseqs.partitions.raxml -m MF + MERGE -T 10 --rcluster 10 -B 1000).

### Comparative genetics

A range of species from within (*n* = 9) and outside (*n* = 5) of the notothenioid clade were chosen. OrthoFinder 2.5.4 was used to identify orthologs present across the different species [96], as well as provide a phylogenetic tree based on common orthologs shared across the different taxa, calculate various statistics, and identify gene duplication events.

### Assembly of the mitochondrial genome

The mitochondrial genome was initially assembled from Illumina reads using GetOrganelle 1.7.5 (-F animal_mt -R 15 --target-genome-size 19,000) [97]. PacBio reads were mapped to the initial assembly using minimap 2.20-r1061 (default parameters), with mapped reads identified using Samtools 1.15, and output to a separate file using Seqtk 1.3-r106. Canu 2.2 was used to assemble the mitochondrial genome using PacBio reads (default parameters and genomeSize = 20 kb corOutCoverage = 10,000) [60]. Following assembly with Canu, polishing was done using Pilon 1.24 (parameters used: --fix bases), with an alignment of Illumina reads against the assembly using minimap 2.20-r1061 being used as input (default parameters) [61, 62]. The mitochondrial genome was then annotated using the mitos2 webserver [31].

### Identification of differentially expressed transcripts

RSEM 1.3.1 rsem-prepare-reference command [98] was used to index the reference transcriptome (consisting of all predicted genes identified by Funannotate) and rsem-calculate-expression was used to calculate expected gene expression levels using STAR 2.7.10a (--paired-end --strandedness reverse --star-gzipped-read-file --star) [99]. The rsem-generate-data-matrix command was then used to combine results from across samples. EdgeR 3.36.0 package [100] in R 4.1.2 [101] was used to compare and detect gene expression level variations across different tissues. For each tissue, expected gene expression levels were imported into R 4.1.2 [101] and the DGEList function was used to convert the results into a format suitable for use with the edgeR 3.36.0 package [100]. Low abundance transcripts were filtered out with filterByExpr function (default parameters), calcNormFactors function was used to calculate data scaling factors for the different libraries and differentially expressed genes between two experimental groups (each tissue vs. all other tissues pooled together) were identified using the exactTest function (square root dispersion value = 0.4). The gene lists were then ranked by log2-fold change in gene expression between the two experimental groups and used to carry out a gene set enrichment analysis (GSEA) of GO using the gseGo function from the clusterProfiler 4.2.2 package [102], with default parameters and a custom database with gene ontologies inferred using InterProScan and Funannotate (converted into an appropriate format using the AnnotationForge 1.36.0 package [103]). Transcripts were considered to be expressed in a tissue when expected read counts were ≥ 10 (as determined by RSEM).

### Identification/characterisation of the AFGP locus

FLNC reads from the pooled IsoSeq3 library and Illumina reads from individual tissues were aligned against the Antarctic toothfish AFGP transcripts (NCBI Genbank Accession HQ447059.1) using the map-hifi and sr alignment profiles respectively (minimap 2.20) [61, 62]. Coverage was calculated using Samtools 1.15 to determine which transcripts in the AFGP locus for Antarctic toothfish were being actively expressed within Patagonian toothfish.

The draft genome assembly, including both primary contigs and haplotigs, was aligned against the publicly available haplotypes for the AFGP locus in Antarctic toothfish (HQ447059.1 and HQ447060.1) using nucmer 4.0.0rc1 [104]. Alignments with > 90% identity and longer than 1000 bp were identified. Regions of interest were checked for presence of *hsl* and *tomm40* genes, which have previously been identified as being situated at the 5’ and 3’ end of the AFGP locus [19]. These genes were cross referenced against OrthoFinder results to check orthology/paralogy and to confirm that corresponding sequences in the assembled genomes of species such as *C. gobio* and *P. georgianus* included similar genetic elements such as trypsin, peptidase and AFGP genes. PacBio reads were mapped to the candidate AFGP loci using minimap 2.20-r1061 and the map-pb preset. Samtools 1.15 was used to exclude alignments shorter than 10 kb in length relative to the reference.

Blastn was used to identify regions of similarity between the AFGP loci of Patagonian toothfish (scaffold_69; position 226,982 − 461,816) and Antarctic toothfish (HQ447059.1; position 516 − 438,650). A schematic representation showing the annotated AFGP loci of the two species of toothfish alongside that of the same locus in the *C. gobio* genome (NC_041370.1; position 3,945,475–4,065,448) was created using gggenes v0.4.1 (https://wilkox.org/gggenes/).

### Electronic supplementary material

Below is the link to the electronic supplementary material.

Supplementary Material 1

Supplementary Material 2

Supplementary Material 3

## Data Availability

All raw data, genome assembly and annotations have been deposited to the National Center for Biotechnology Information (NCBI) databases under BioProject PRJNA864592 and Biosamples SAMN30075165 and SAMN30114550-SAMN30114559. The Whole Genome Shotgun project has been deposited at DDBJ/ENA/GenBank under the accession JAOVFM000000000. The version described in this paper is version JAOVFM010000000.
